# “Rapid Tele-Psychotherapy” With Single-Session Music Therapy for a Broken-Hearted Girl With Hopelessness, Depression, and Suicidal Ideation: A Case Report

**DOI:** 10.1155/crps/5525861

**Published:** 2025-11-25

**Authors:** Dominikus David Biondi Situmorang

**Affiliations:** ^1^Department of Guidance and Counseling, Faculty of Education and Languages, Atma Jaya Catholic University of Indonesia, Jl. Jenderal Sudirman 51, DKI Jakarta 12930, Indonesia; ^2^Doctoral Program in Guidance and Counseling, Faculty of Education, Universitas Pendidikan Indonesia, Jl. Dr. Setiabudhi No. 229, Bandung 40154, Indonesia

**Keywords:** broken-hearted, depression, hopelessness, mental health services, rapid tele-psychotherapy, single-session music therapy, suicidal ideation

## Abstract

**Objective:**

In the wider context of palliative care, problems faced by young people today, such as depression, mental illness, and suicide; are considered terminal problems, so they must be treated immediately and given appropriate intervention. Caution is needed in providing appropriate interventions for them, of course, interventions that are by their characteristics. Based on their character, they are a generation that is quite fragile and easily discouraged; on the other hand, they are a generation that is dynamic and passionate about creativity. There needs to be a match between what they need and what the intervention provides. One type of intervention in palliative care that has recently been offered is “rapid tele-psychotherapy” with single-session music therapy (RTP-SSMT), which is considered quite appropriate in responding to these challenges.

**Method:**

I report the case of a 22-year-old broken-hearted girl with hopelessness, depression, and suicidal ideation.

**Results:**

I describe the effectiveness of the implementation of RTP-SSMT in reducing the scales of hopelessness, depression, and suicidal ideation.

**Significance of Results:**

It can be concluded that the implementation of the RTP-SSMT for a broken-hearted girl with conditions of hopelessness, depression, and suicidal ideation who can be at risk for suicide can be said to be effective in alleviating these negative feelings. Aside from that, through this study, the biggest implication is that the RTP-SSMT intervention theory can be a choice for mental health workers who want to process assistance to patients/clients who experience hopelessness, depression, and suicidal ideation in someone who has experienced a breakup.

**Summary**

• What is already known on this topic:
  ⚬ A psychotherapeutic approach as a creative and responsive intervention for young people with different characters nowadays is still very rare. This is considered very important to test the effectiveness and efficacy of a new approach for young people who experience broken-heartedness with hopelessness, depression, and suicidal ideation by using “rapid tele-psychotherapy” with single-session music therapy (RTP-SSMT).
• What this study adds:
  ⚬ This study is very important to provide new insight that there is a new intervention approach that has been proven effective in alleviating problems that are often encountered in today's youth.
• How this study might affect research, practice, or policy:
  ⚬ Through this study, the biggest implication is that the RTP-SSMT intervention theory can be a choice for mental health workers who want to process assistance to patients/clients who experience hopelessness, depression, and suicidal ideation in someone who has experienced a breakup.

## 1. Introduction

Nowadays, the number of suicides among young people is increasing [1–3]. There are quite several life problems behind this, one of which is a breakup [4]. Usually, the feelings that arise when experiencing an event like this are hopelessness, depression, and suicidal ideation [5]. If not addressed immediately, of course, it will lead to a decision to commit suicide on them [6].

In the broader context of palliative care, the problems faced by young people today, such as depression, mental illness, and suicide; are considered terminal ones [7], so they must immediately be treated and given appropriate intervention. Care is needed in providing the right intervention for them, of course, an intervention that is suitable for their characteristics. Based on their character, they are a generation that is quite fragile and easily discouraged [8, 9]. However, on the other hand, they are a dynamic generation and are passionate about creativity [10–12]. So, in this case, there needs to be a match between what they need and what the intervention provides.

One type of intervention in palliative care that has recently been offered is “rapid tele-psychotherapy” with single-session music therapy (RTP-SSMT), which is considered quite appropriate in responding to these challenges [13]. In its development, this intervention has been tested for its effectiveness on one of the COVID-19 patients [14] and won a world record award [15]. In addition, this intervention is recommended to be implemented in the Metaverse in the future [16].

Furthermore, in this article, the author reports on the effectiveness of implementing RTP-SSMT to a late adolescent girl with conditions of hopelessness, depression, and suicidal ideation who can be at risk for suicide, where this intervention can be claimed to be effective in reducing negative feelings.

## 2. Case Report

**Figure 1:**
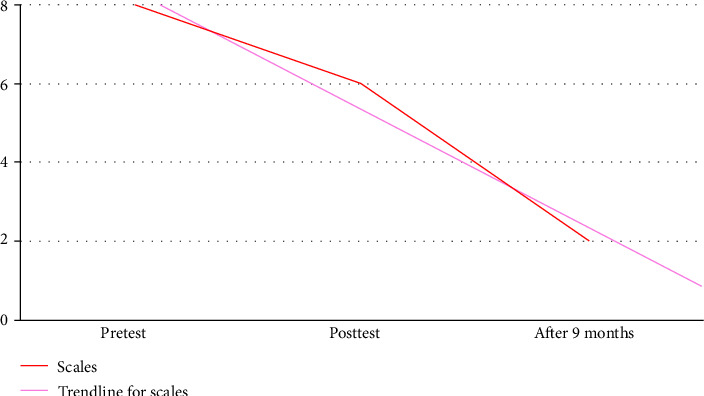
Level of hopelessness, depression, and suicidal ideation.

**Figure 2:**
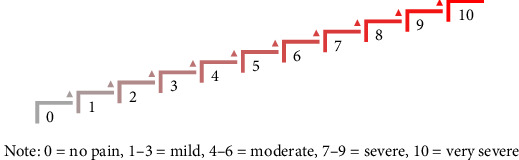
Psychological problems scales of “scaling questions”. 0 = no pain, 1–3 = mild, 4–6 = moderate, 7–9 = severe, 10 = very severe.

**Figure 3:**
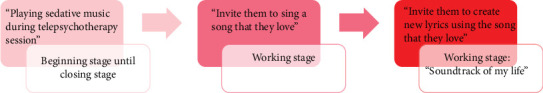
Three keys' strategies of “rapid tele-psychotherapy” with SSMT.

A 22-year-old girl has a broken heart problem. She admitted to experiencing hopelessness, depression, and suicidal ideation after breaking up with her boyfriend. She broke up with her boyfriend because she was being cheated on, and until now she has always failed to move on. Every time she wants to do her daily activities, she is not excited and always remembers all the events that have been experienced with her lover. She felt hopeless, and her life had no meaning anymore, because she loved her lover very much. They both had promised to get married one day, but the promise ran aground because of the presence of another ideal woman in her lover's heart. During the implementation of the RTP-SSMT, she gave a fairly high scale of hopelessness, depression, and suicidal ideation, represented by a score of 8 on the scaling questions given (Figures 1 and 2). During the implementation of the working stage, especially when giving miracle questions, she gave quite good answers and was able to participate well in the implementation of two strategies, namely *“invite them to sing a song that they love”* and *“invite them to create new lyrics using the song that they love”* (Figure 3). In the closing stages, she was able to compose new lyrics of the song that she liked, which she made the *“soundtrack of my life.”* At the end of the session, she admitted that hopelessness, depression, and suicidal ideation had dropped to 6 (Figures 1 and 2). Furthermore, she admitted that after 9 months of implementing the RTP-SSMT, the level of various problems experienced by her had dropped to a score of 2 (Figures 1 and 2).

## 3. Discussion

In this case, there is a difference in the effectiveness of reducing negative feelings from the previous case [13]. In the case of the patient/client who experienced anxiety, panic, fear, depression, acute stress, insomnia, and delusions of death, the scale decreased from 10 to 5 as measured by scaling questions [13, 14]. However, in this case, the decrease in feelings of hopelessness, depression, and suicidal ideation is only 2 scores, namely from 8 to 6 (Figures 1 and 2). It can be identified that everyone from different problem backgrounds will experience different impacts from the application of the RTP-SSMT. Of course, this is also due to the differences in beliefs and values that everyone has [17, 18]. Coupled with this is the ability of a person to take meaning from every event in their life, even in pain [19, 20]. However, after 9 months, she admitted that her current level of various problems was at number 2 (Figures 1 and 2). She said that for 9 months after carrying out the RTP-SSMT intervention, she felt that her life had changed as if she had a “new life;” she no longer had negative feelings, and she no longer thought about the problems she faced yesterday. Now she can focus on herself, and she has also got a new job.

At the beginning stage of the RTP-SSMT implementation, the patient/client is asked to choose a song that can represent her current feelings as an illustration of presenting problems as well as rapport building. She chose the song *“Sisa Rasa”* from Mahalini (a female singer from Indonesia). She admitted that this song represents her current feelings. In this section, the patient/client reveals that she is heartbroken. After that, she was given informed consent as a form of approval for the implementation of the RTP-SSMT. Then, sedative music is played so that the patient/client can feel relaxed and calmer during the RTP-SSMT session as the implementation of *“playing sedative music during the rapid tele-psychotherapy session”* (Figure 3). At the closing of this stage, the patient/client is given scaling questions [21] as a pretest to describe her negative feelings. She claimed to experience hopelessness, depression, and suicidal ideation at a score of 8 (Figures 1 and 2). The patient/client admitted that previously she could judge that she was at a score of 10, but she managed to reduce 2 points (Figures 1 and 2), because she had done the counseling process many times with her colleagues who had counseling backgrounds. She confessed that by telling the person over and over again, she experienced some relief but not full. So, she needs further assistance from mental health professionals. In this context, it can be said that she had a “talking cure,” that just by telling stories she experienced healing [22, 23].

Furthermore, at the transition stage, the patient/client has explained the procedures that will be carried out during the RTP-SSMT. In addition, she was asked to be able to set the goals of this session. It is important to do this so that the patient/client and mental health workers jointly reach an agreement in achieving common goals in the counseling process, especially for alleviating patient/client problems [24]. In this section, she sets a goal so that she can let her lover go, forgive him, move on, and become a new person.

**Figure 4:**
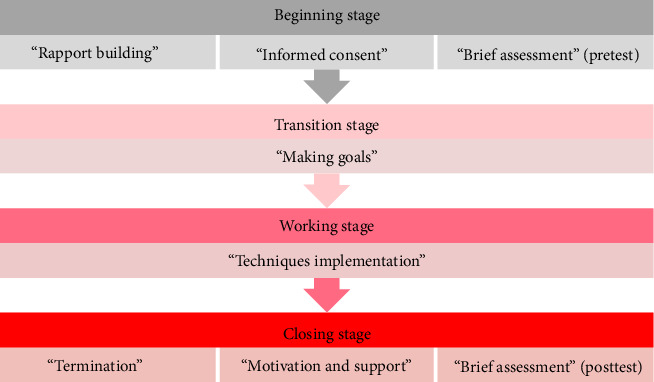
The stages of “rapid tele-psychotherapy” with SSMT.

At the working stage, the patient/client is given miracle questions [25] to help her gain new insights about what she should do next, so that she can become the person mentioned in the previous stage and can be happier than before (Figure 4). She said that she had to change her mindset, change her lifestyle, try to solve her problems, increase her prayer routine, and the patient/client demonstrated increased participation in meaningful social and spiritual activities, which she associated with improved well-being. After that, she was invited to carry out an *“invite them to sing a song that they love”* (Figure 3). In this part, she was asked to sing a song that she liked, then she reselected the song she had chosen at the beginning of the previous stage. After that, she was invited to carry out an *“invite them to create new lyrics using the song that they love”* (Figure 3). In this part, she changes the content of the song's lyrics to be more positive and motivates herself to rise from her broken heart. Especially in this part, there are lyrics that she created containing positive sentences that inspire her to move on, namely: *“You must be replaced by someone else, I will survive. God, I will let him go to her!”*. After composing the lyrics of the song and singing it, she feels more relieved, sincere, and she can let go of her lover so that he can live happily with other women. In this part, it can be claimed that she engaged in music-based reframing by composing personalized lyrics, which served as a coping mechanism and self-affirmation tool; it's called the *“soundtrack of my life”* that she can interpret and remember for a lifetime (Figure 3). If it is associated with the theory of logotherapy [20] and meaning-centered [19], the patient/client has the meaning of the sad event that she has experienced. She can also interpret the event as a moment that makes her become a stronger person. In addition, from a spiritual point of view, she wants to be closer to God by increasing her routine in prayer and chooses to become a servant of God by becoming a Sunday school teacher at her church.

Last but not least, at the closing stage, the patient/client is asked about what insights have been received during the RTP-SSMT process. She admitted that she experienced relief from the process that had been carried out. In this part, she is given motivation and support that she is a person who can construct solutions to her problems, so it is hoped that she will be more confident and independent in solving her problems after this session is completed. She is motivated to be the best version of herself [26]. After that, she was reminded again that when one day she experiences the same problem again, please sing the *“soundtrack of my life”* that was created on the previous working stage (Figure 3). Of course, the song will be an encouragement when she experiences her problems again in the future. In closing, she was given scaling questions [21] as a posttest to determine the changes in negative feelings that occurred in her. She admitted that she experienced a decrease in hopelessness, depression, and suicidal ideation at a score of 6 (Figures 1 and 2). This score will continue to decrease gradually, if the patient can build a new habit [27] which has been formed from the commitments that she has made through her new core beliefs [28]. It is evident that 9 months later, she has a score of 2 for the various problems that she experienced (Figures 1 and 2).

Furthermore, scaling (0–10) has been used in counseling psychology for many years as a brief subjective measure to gather clients' self-reported changes [21]. They are not equivalent to standardized psychometric instruments but are clinically efficient in evaluating immediate therapeutic impacts. However, future research should use validated scales and triangulation to enhance the evidence. The scores of the patient decreased from 8 to 6 and then to 2 at 9 months; however, their clinical significance needs be considered with caution. The intervention may have been a guide for immediate symptom reduction, but the influence of external context variables and aspects of their working life (e.g., employment and religious engagement) is also probably of importance.

When interpreting findings of the present case also one should keep in mind what numerical reduction translates into as clinically meaningful improvement. The significant reduction in the patient's self-rated hopelessness, depression, and suicidal intent from 8 on this visit to 6 after the intervention, and then down further to 2 in 9 months later, reflects a dramatic subjective response. In clinical practice, a two-point or larger reduction in the 0–10 distress rating score is generally considered to be meaningful for patients because it represents a change from severe to moderate or mild levels of distress [21]. In addition, Jacobson and Truax's [29] criteria for clinically meaningful change include a shift in patient functioning from the dysfunctional to functional range, as well as *“the consistency of this change across different areas of dysfunction,”* and is a change beyond that due to chance or noise. From this point of view, the course of our patient reflects consistent and clinically significant improvement over time.

However, it is too early to credit this outcome just at the foot of the intervention. The healing session may have acted as a priming event by promoting the use of coping mechanisms and reinterpreting symptoms [30], whereas ongoing life circumstances—like working and attending church or social activities—probably further bolstered recovery. Consequently, although the clinical meaningfulness of this level of gain is clear from subjective and theoretical perspectives, interpretation should be guarded, and studies with controlled serial designs using established instruments are required to verify the standalone effectiveness of rapid tele-psychotherapy through single-session music therapy.

## 4. Conclusion

Based on the case report described above, it can be concluded that the implementation of the RTP-SSMT for a broken-hearted girl with conditions of hopelessness, depression, and suicidal ideation who can be at risk for suicide can be said to be effective in alleviating these negative feelings. The RTP-SSMT has proven that this approach can empower individuals to fight independently in alleviating her problems.

## 5. Limitations and Suggestions

The present study has some limitations that should be recognized. It was a single nonvoluntarily-referred patient that initiated the case. The lack of a formal means of selecting patients for this intervention could potentially lead to convenience bias, and it is important to note that the patient had previously received *ad hoc* counseling support from colleagues; this may have facilitated her willingness to engage with the intervention.

Second, in the 9 months post-intervention, the participant made significant external changes: finding new employment and becoming more involved in religious activities. These contextual changes were not excluded and may have indeed acted as major confounders with respect to decreased hopelessness, depressive symptoms, and suicidal ideation. Culture and gender factors (which are important; especially in the Indonesian context, where sociocultural norms play a significant role in shaping what romantic breakup means emotionally) were not considered. These constraints affect the transferability of results to other populations and cultures.

Third, the intervention effects were evaluated on only subjective 0–10 scale questions. Although this method gave valuable insights into the self-reported changes of the patient, standardized and validated instruments were not applied, and further, no third-party (e.g., family members or mental health care professionals) triangulation was made. This raises the possibility of response bias, such as social desirability and the impact of the therapeutic relationship.

Fourth, the placebo effect on the therapeutic attention itself cannot be eliminated. Additionally, the lack of an arranged and standardized intervention approach may also render it difficult to be replicated by other professionals. This is especially true when considering a specific effect of music therapy in the single-session tele-psychotherapy.

Finally, practical constraints need to be considered. This intervention is feasible, provided successful implementation would be both music therapy skills and access to suitable teleconsultation applications. The potential for and use of such an approach is probably limited in low-resource or rural environments, where barriers to engaging may include internet availability and difficulties with maintaining confidentiality during tele-sessions.

The present study had several limitations, and findings indicated the need for follow-up studies with more rigorous designs. To improve the evidence of evidence, comparative groups, mixed-methods designs, and validated measures with systematic control for contextual variables are required. Also, cultural adaptation warrants exploration to ensure the cultural adaptations of intervention for people from multiple ethnic backgrounds. Aside from that, it is hoped that the patient/client can grow into an independent person after this session by continuing to carry out the commitments she has made before. In addition, it is recommended that the *“soundtrack of my life”* that has been created can be used as a lifelong reminder that through this event she can grow into a greater person than before.

## 6. Implications

Through this study, the biggest implication is that the RTP-SSMT intervention theory can be a choice for mental health workers who want to process assistance to patients/clients who experience hopelessness, depression, and suicidal ideation in someone who has experienced a breakup.

## 7. Direction of Future Research

The RTP-SSMT intervention will continue to evolve. To test the efficacy, efficiency, and effectiveness of this theory, it should also be applied to other mental health problems. In addition, it is also suggested that it can be applied to a group atmosphere so that the application of this theory becomes wider in scope. In other words, it can be studied again based on the factors of age, gender, religion, and cultural differences.

## Data Availability

The data that support the findings of this study are available upon request from the corresponding author.

## General Statement

Validated scales or measures were used to assess depression, hopelessness, and suicidal ideation before and after the intervention:

The author used “Scaling Questions” from 0 to 10 (0 = no pain, 1–3 = mild, 4–6 = moderate, 7–9 = severe, 10 = very severe), which describe hopelessness, depression, and suicidal ideation. These questions were verbal and recorded by the author, adapting concepts that have been published in the article: [13].
